# Acupuncture as adjunctive therapy for patients with AECOPD: study protocol for a multicenter, randomized controlled trial

**DOI:** 10.3389/fpubh.2023.1235672

**Published:** 2023-10-02

**Authors:** Chunyan Yang, Mingsheng Sun, Guixing Xu, Qin Luo, Liuyang Huang, Hao Tian, Siyao Gong, Qian Li, Xin Yu, Ming Chen, Dan Huang, Yilin Liu, Zhuo Zhou, Fengyuan Huang, Yunyu Liu, Juan Tang, Sha Yang, Fang Zeng, Fanrong Liang

**Affiliations:** Acupuncture and Tuina School, Chengdu University of Traditional Chinese Medicine, Chengdu, Sichuan, China

**Keywords:** acute exacerbation of chronic obstructive pulmonary disease, acupuncture, acupoint combinations, randomized controlled trial, study protocol

## Abstract

**Background:**

The acute exacerbation of chronic obstructive pulmonary disease (AECOPD) is a common respiratory disease among older adults, which imposes a significant burden on individuals and society and poses a major challenge to the global public health system due to its high morbidity and mortality. Acupuncture is effective for AECOPD, but its efficacy has been questioned due to the limited methodological quality. Thus, we aim to investigate the efficacy of acupuncture as adjunctive therapy for AECOPD and determine whether the efficacy of acupuncture differs with the type of acupoint combinations.

**Methods and analysis:**

This study proposes a prospective, multicenter randomized controlled trial that will comprise four groups, including two acupuncture treatment groups, one sham acupuncture group, and one basic treatment group. The acupuncture treatment groups will be distinguished by their focus on different patterns of acupoint combination, namely the Xi-cleft and He-sea acupoint combination and the Eight Confluence points acupoint combination, which may vary in clinical efficacy based on traditional acupuncture theories. The study aims to randomize 556 patients in a 1:1:1:1 ratio across the four groups. Each patient in acupuncture group or sham acupuncture group will receive routine drug therapy and 7 sessions of acupuncture treatment over 1 week. Participants in the basic treatment group will only receive routine drug therapy. The trial will be conducted in seven hospitals located in China. The primary outcomes in this trial will include differences in the Breathlessness, Cough, and Sputum Scale (BCSS) before randomization, 7 days after randomization, 5 and 9 weeks after randomization.

**Ethics and dissemination:**

Ethical approval was obtained from the Sichuan Regional Ethics Review of Committee on Traditional Chinese Medicine (Approval ID: 2022KL-068). The results of this study will be distributed through peer-reviewed journals.

**Clinical Trial Registration**: ClinicalTrials.gov, identifier ChiCTR2200064484.

## 1. Introduction

Chronic obstructive pulmonary disease (COPD) is a kind of chronic respiratory disease, characterized by progressive and irreversible airflow obstruction (1). COPD is a major cause of chronic morbidity and mortality worldwide, and many people suffer from this disease and die from it or its complications, making it an important public health challenge (1, 2). The high-risk group of COPD is mainly middle-aged or older adults (aged ≥40 years) (3, 4). As the aging population intensifies globally, the incidence and mortality of COPD will continue to increase, and the COPD burden is projected to increase over the coming decades (5–8). Acute exacerbations of COPD (AECOPD) lead to a dramatic deterioration of lung function and an acute exacerbation of respiratory symptoms, such as cough, dyspnea, sputum production and purulence, requiring additional therapy (1, 9, 10). AECOPD is associated with poor prognosis and increased risk of death (11). Furthermore, AECOPD significantly burdens patients, their families, caregivers, and society, and the cost changes as the disease progresses, which accounts for the greatest proportion of the total COPD burden on the healthcare system (1, 12). Research indicates that COPD patients experience acute exacerbations at a rate of 0.5–3.5 times per year (13, 14), with an associated average hospitalization cost of up to 11,598 RMB per person annually in China (15). Over $50 billion of direct and indirect costs are generated by COPD exacerbations in the US alone per year (1, 16). Steroids, bronchodilators, and antibiotics are common drugs for AECOPD, which have shown obvious therapeutic effects. However, adverse events are also very common, such as diabetes, osteoporosis, cross-infection, and antibiotic resistance (17–19).

A majority of clinical studies (20, 21) and systematic reviews (22–24) have concluded that acupuncture is a safe and potentially effective intervention for COPD patients in the stable phase. However, few studies have examined the effect of acupuncture on AECOPD during hospitalization. In recent years, several studies have paid attention to this field, demonstrating that acupuncture alleviates clinical symptoms (cough, sputum, wheeze, dyspnea, etc.), quality of life, exercise performance and lung function (25–27). However, most of the existing studies were single-center studies, with small sample size, low quality and lack of standardized acupuncture operation procedures and rigorous methodological design (28–30). Consequently, the outcomes of these studies present a challenge in arriving at definitive deductions. As a solution, we have developed a multicenter randomized controlled trial to tackle these issues and furnish more robust substantiation for the efficacy of acupuncture in managing AECOPD. Furthermore, the therapeutic effects of acupuncture may be significantly influenced by various acupoint combinations (31, 32). However, the most effective acupoint combination for treating AECOPD remains uncertain and necessitates further validation.

Thus, the first aim of this trial will focus on the efficacy and safety of acupuncture as an adjunctive treatment to conventional treatment for patients with AECOPD. Second, we will attempt to determine whether the efficacy of acupuncture differs with the type of acupoint combinations.

## 2. Methods and design

### 2.1. Study design

**Figure 1:**
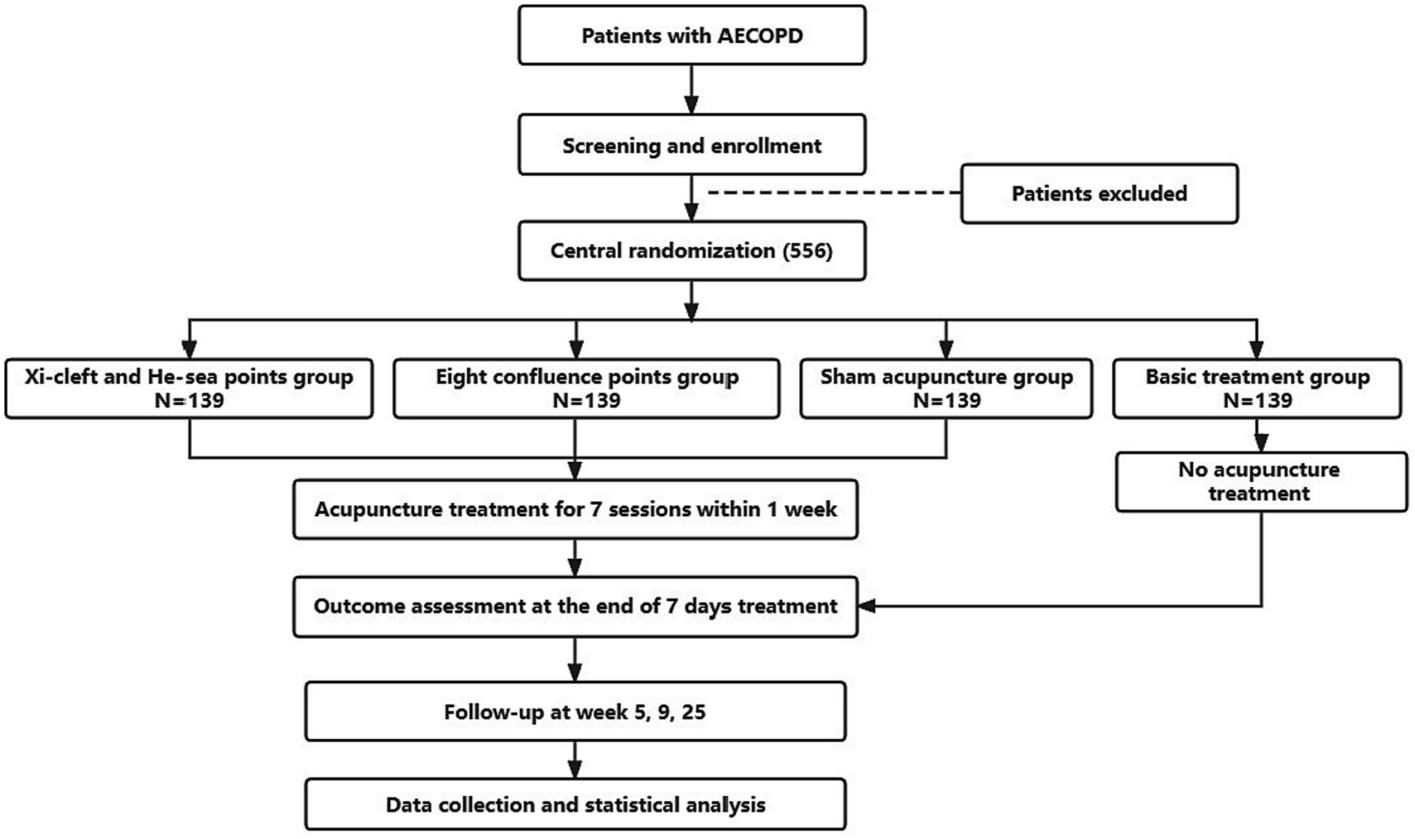
The study flowchart.

This will be a prospective, multicenter, randomized controlled clinical trial, and 556 participants will be randomly assigned to four groups with a 1:1:1:1 allocation ratio. The trial is planned to be conducted from October 2022 to December 2023 in the Department of Respiratory Internal Medicine at 7 hospitals in China: Chengdu Second People’s Hospital, Meishan Hospital of Traditional Chinese Medicine, People’s Hospital of Meishan City, Mianyang Hospital of Traditional Chinese Medicine, the Third Hospital of Mianyang, People’s Hospital of Deyang City, and Guangyuan Central Hospital. The two acupuncture groups (the Xi-cleft and He-sea points group and the Eight Confluence points group) and the sham acupuncture group will continually receive routine drug therapy and 7 sessions of acupuncture treatment (once a day), and the treatment period will last for 1 week. Patients in the basic treatment group will not receive acupuncture treatment during the study period, but routine drug therapy instead. After 25 weeks, they will be given 7 sessions of acupuncture treatment as compensation. This trial will be performed following SPIRIT checklist (33) (Supplementary Table S1). The study flowchart is shown in Figure 1, and the timetable for enrollment, intervention and evaluation is shown in Table 1.

**Table 1:** The schedule of enrollment, interventions, and assessments.

	Enrollment	Allocation	Treatment	Discharge	Follow-up		
Timepoint	−1d	0d	1d–7d		5w	9w	25w
Inclusion and exclusion	×						
Sign the informed consent	×						
Randomization		×					
Interventions							
Xi-cleft and He-sea points group			7 sessions of acupuncture				
Eight confluence points group							
Sham acupuncture group							
Basic treatment group			No acupuncture				
Assessment							
BCSS		×	×		×	×	
PFT	×		×				
ABG		×	×				
CAT		×	×		×	×	
6MWT		×	×				
Hospital length of stay				×			
The number of exacerbations							×
Blindness assessment			×				
Patient’s compliance			×	×	×	×	×
Adverse events			×	×	×	×	×

d, day; w, week; BCSS, breathlessness, cough, and sputum scale; CAT, COPD assessment test; 6MWT, 6-min walking test; PFT, pulmonary function test; ABG, arterial blood gas.

### 2.2. Participants

#### 2.2.1. Diagnostic criteria

According to the 2022 Global Initiative for Chronic Obstructive Pulmonary Disease (GOLD) (1), AECOPD can be diagnosed by: (1) patients who have dyspnea, chronic cough or sputum production, and/or a history of exposure to risk factors for the disease (such as smoking, biofuel exposure and air pollution), (2) spirometry is required to make the diagnosis: a post-bronchodilator FEV1/FVC < 0.70, (3) worsening of respiratory symptoms, such as increased dyspnea, increased sputum purulence and volume, and increased cough and wheeze, (4) ruling out other comorbidities that may worsen respiratory symptoms, such as pneumothorax, pleural effusion and pulmonary embolism.

#### 2.2.2. Inclusion criteria

Participants who meet all the following conditions will be considered for registration. The inclusion criteria will be: (1) patients meet a diagnosis of AECOPD according to GOLD 2022, (2) males or females between 40 and 80 years of age, (3) the classification of pulmonary function is at the level of moderate, severe or extremely severe (FEV_1_ < 80%), (4) patients who agree to participate in the study voluntarily and sign the informed consent form.

#### 2.2.3. Exclusion criteria

Patients with any one of the following criteria will be excluded: (1) Patients with severe cardiovascular and cerebrovascular, neurological, blood system or immune system diseases, or malignant tumors. (2) Patients with respiratory system diseases that have an obvious impact on this research, such as active pulmonary tuberculosis, bronchial asthma, severe bronchiectasis, pulmonary atelectasis, primary pulmonary hypertension, pulmonary interstitial diseases. (3) Patients with a history of lung surgery, such as segmentectomy, wedge resection, lobectomy, and pneumonectomy. (4) Patients have difficulty answering the questionnaires because of consciousness disorder, psychiatric disease or severe cognitive dysfunction. (5) Patients are undergoing noninvasive mechanical ventilation or trachea intubation. (6) Patients who have accepted acupuncture treatment due to respiratory systems diseases in the past 1 month. (7) Patients are pregnant, breastfeeding or planning a pregnancy during the study period. (8) Patients are participating in other clinical trials.

### 2.3. Recruitment process

Participants will be recruited from the Department of Respiratory Internal Medicine in 7 hospitals in China: Chengdu Second People’s Hospital, Meishan Hospital of Traditional Chinese Medicine, People’s Hospital of Meishan City, Mianyang Hospital of Traditional Chinese Medicine, the Third Hospital of Mianyang, People’s Hospital of Deyang City, and Guangyuan Central Hospital. Research assistants will be sent to help screen participants in these hospitals. Besides, participants will be recruited through posting posters at respiratory wards.

### 2.4. Written informed consent

Before randomization, all patients will be informed of the allocation, possible benefits and risks of participating in this trial. If they are willing to participate in this study, then they will sign the written informed consent. The patients could refuse to participate in the study or withdraw from the study at any time during the study, without a specific reason and any penalty or loss of benefits. Patients will be required to give their phone numbers, so that the researchers can maintain close contact with the participants and improve patients’ compliance.

### 2.5. Patient adherence

In order to ensure the completion of the trial, efforts will be made to ensure patient compliance. First, we will fully learn knowledge about AECOPD before the study and fully reflect our professionalism in communication, in order to enhance patient trust in us. Second, we will inform patients that acupuncture treatment is performed on the basis of conventional treatment and will not endanger their health, and objectively explain the manipulation, clinical efficacy, safety and possible mechanisms of acupuncture to patients, so as to dispel the patient’s concerns. Third, acupuncturists will receive professional training to improve their proficiency, so as to ensure that patients get the best acupuncture experience. Besides, we also maintain good communication with the patient’s family, so that they can fully understand the patient’s condition, and urge the patient to better accept acupuncture treatment. And all tests (such as arterial blood gas, pulmonary function test), evaluations and acupuncture treatment involved in the trials are free of charge. Patients in basic treatment group will receive 7 free acupuncture treatments as compensation at the end of the follow-up period. And the drop outs and reasons will be recorded in the observation and follow up period.

### 2.6. Randomization, allocation concealment, and blinding

The study will use central randomization which can be completed using a computer-generated random allocation sequence by Chinese Evidence-based Medicine Center, West China Hospital, Sichuan University. Research assistant in each hospital will be registered in the randomization center (located in Brightech-Magnsoft Data Services Company) and trained to apply for randomization through the online website. And they will obtain randomization sequence and group assignments through the internet system or E-mail message. The randomization sequence will be concealed in the server of Chinese Evidence-based Medicine Center, West China Hospital, Sichuan University until this study finishes participant enrollment, observation and data collection.

In this study, we will apply sham acupuncture at non-acupoints to mask verum and sham acupuncture between patients from the verum and sham acupuncture treatment groups. However, because of the nature of acupuncture, participants in the basic treatment group and acupuncturists will not be blinded. At the same time, each patient will be treated in a separate room to ensure that there is no opportunity for communication. And when participants complete all 7 sessions of acupuncture treatment, they will be asked whether they have accepted verum acupuncture or sham acupuncture to assess the blinding effects. The efficacy evaluators and statisticians will be separated and blinded.

### 2.7. Interventions

The treatment plan is guided by acupuncture and traditional Chinese medicine (TCM) theory, and developed through previous literature research and consensus of acupuncture experts. There are four groups in this study: two acupuncture groups (the Xi-cleft and He-sea points group, and the Eight Confluence points group) and two control groups (the sham acupuncture group and the basic treatment group). Patients in these four groups are required to accept conventional medical treatment recommended by GOLD 2022 (1). Based on the revised STRICTA recommendations (34), detailed information on acupuncture treatment is summarized in Supplementary Table S2. And these acupoints and non-acupoints will be punctured with disposable stainless needles (0.25 mm × 13 mm, 0.25 mm × 25 mm; Huatuo Suzhou, China). The position of the aforementioned acupoints will be based on the nomenclature and location of acupuncture points designed by the National Standard of the People’s Republic of China (GB/T 12346–2021) (35). Acupuncture treatment will be performed by acupuncturists who hold a Chinese medicine practice license issued by the Ministry of Health of the People’s Republic of China, and have acupuncture clinical practice experience of more than 3 years.

#### 2.7.1. Acupuncture groups

**Figure 2:**
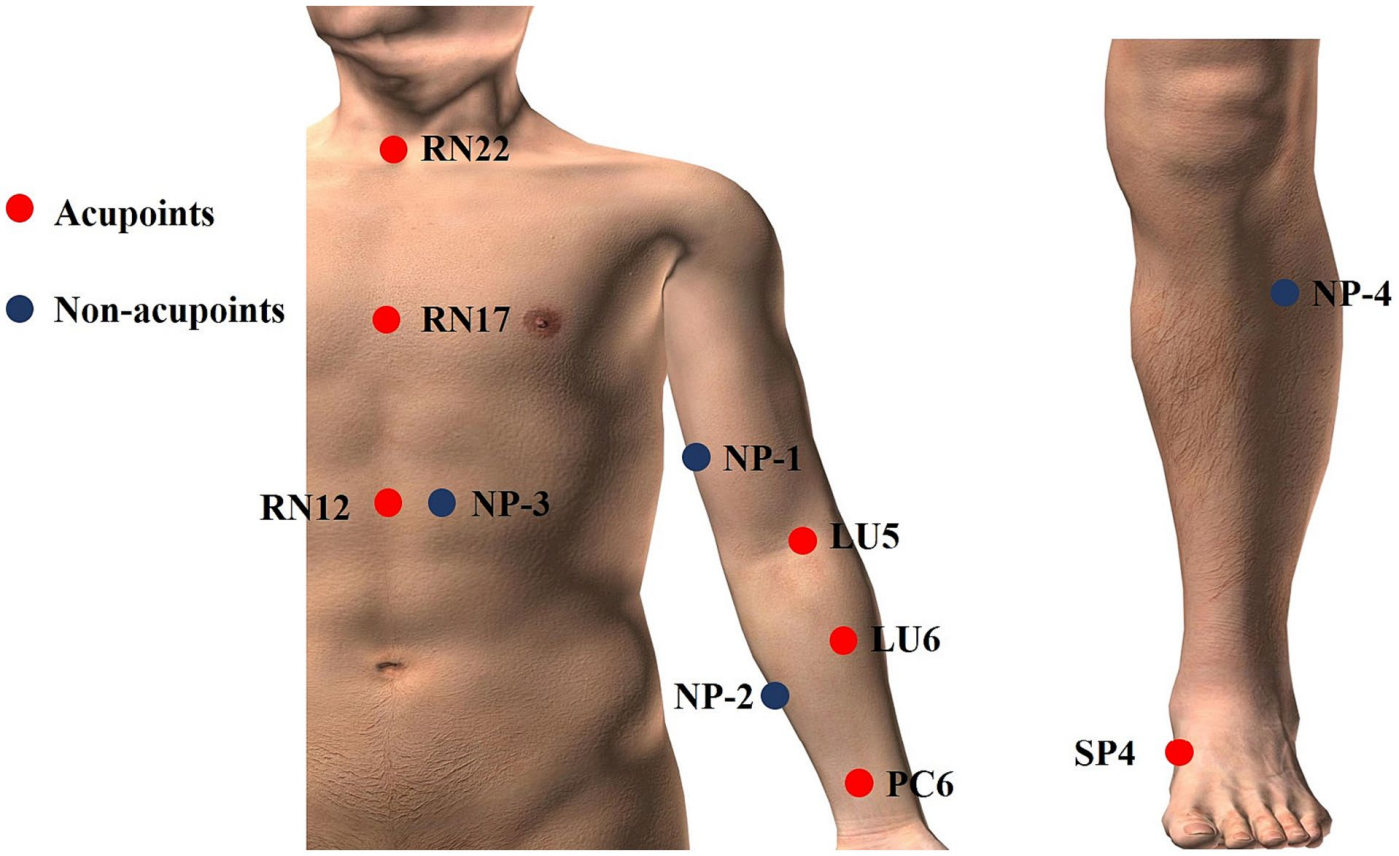
Locations of acupoints and non-acupoints.

There are two acupuncture groups with different acupoint combinations: one is Xi-cleft and He-sea points group, the other is the Eight Confluence points group. The treatment plan is based on previous literature research (32) and consensus with acupuncture experts. We select Tiantu (RN22), Zhongwan (RN12), and Danzhong (RN17) as the basic acupoint prescription in the two verum acupuncture groups. Basic acupoints will be punctured with 0.25 mm × 13 mm acupuncture needles. Operation methods are as follows: Tiantu (RN22) will be punctured perpendicularly 0.2–0.3 cun, and then, make the needle downwards, and punctured 0.5 cun close to the rear of the manubrium sternum. Zhongwan (RN12) will be punctured perpendicularly 0.3–0.5 cun. Danzhong (RN17) will be punctured transversely 0.3–0.5 cun. And then, we will use two different acupoint combination patterns to explore whether there are differences between different acupoint combinations. Grouping and acupoints are as follows. And the location of acupoints is displayed in Table 2 and Figure 2.

**Table 2:** Acupoints used in the acupuncture group.

Acupoint	Location
RN22 (Tiantu)	On the anterior midline, in the center of the suprasternal fossa.
RN17 (Danzhong)	On the anterior midline, at the level of the fourth intercostal space, at the midpoint of the two nipples
RN12 (Zhongwan)	On the anterior midline, 4 cun above the umbilicus.
LU5 (Chize)	On the cubital crease, the radial side of the tendon of biceps brachii (needled bilaterally)
LU6 (Kongzui)	On the line joining Chize (LU5) and Taiyuan (LU9), 7 cun above the transverse crease of the wrist (needled bilaterally)
PC6 (Neiguan)	2 cun above the transverse crease of the wrist, on the line connecting Quze (PC3) and Daling (PC7), between the tendons of palmaris longus and flexor carpi radialis (needled bilaterally)
SP4 (Gongsun)	On the medial aspect of the foot, in the depression distal and inferior to the base of the first metatarsal bone (needled bilaterally)

##### 2.7.1.1. Xi-cleft and He-sea points group

Except for the three basic points, Kongzui (LU6) and Chize (LU5) are selected in this group, which are the Xi-cleft point and He-sea point of Lung Meridian of Hand-Taiyin, respectively. These two points will be punctured perpendicularly 0.8–1 cun with 0.25 mm × 25 mm acupuncture needles.

##### 2.7.1.2. Eight-confluent points group

Based on the basic acupoints, Neiguan (PC6) and Gongsun (SP4) are selected in this group, both of which are the Eight-confluent points. These two points will be punctured perpendicularly 0.8–1 cun with 0.25 mm × 25 mm acupuncture needles.

And the needles will be manipulated with lifting, thrusting, twirling and rotating after inserting to achieve Deqi (a sensation including soreness, numbness, distention and heaviness). The treatment will last 30 min, and during the needle retention period, the needles will be manipulated by lifting, thrusting, twirling and rotating every 15 min to maintain the Deqi sensation. Every participant will receive acupuncture treatment once a day for a total of 7 sessions continuously.

#### 2.7.2. Control groups

##### 2.7.2.1. Sham acupuncture group

Participants assigned randomly to this group will be given superficial puncturing at non-acupoints. Acupuncture needles will be inserted at four bilateral non-acupoints in terms of previous research (36) and expert proposes. The non-acupoints will be punctured superficially 0.1–0.3 cun with 0.25 mm × 13 mm acupuncture needles. Participants in the sham acupuncture group will also receive the same stimulation and the same duration of treatments as the participants received in the acupuncture group, but without achieving a Deqi sensation. And the location of non-acupoints is displayed in Table 3 and Figure 2.

**Table 3:** Non-acupoints used in the sham acupuncture group.

Non-acupoint	Location
Non-acupoint 1 (NP-1)	On the medial anterior border of the upper arm, half between the tip of the elbow and the axilla (needled bilaterally)
Non-acupoint 2 (NP-2)	On the medial anterior border of the forearm, half between epicondyles medialis of the humerus and ulnar side of the wrist (needled bilaterally)
Non-acupoint 3 (NP-3)	On the middle of abdomen, 1.25 cun lateral to RN12 (Zhongwan), midpoint of Stomach Meridian and Kidney Meridian (needled bilaterally)
Non-acupoint 4 (NP-4)	On the lateral side of the lower leg, 1 cun lateral to ST36 (Zusanli), between Stomach Meridian and Gallbladder Meridia (needled bilaterally)

##### 2.7.2.2. Basic treatment group

Participants will receive no acupuncture treatment in the basic treatment group, but receive the conventional treatment recommended by GOLD. And patients in this group will subsequently have the option of 1 week (7 sessions) acupuncture treatment for free at the end of the follow-up period.

#### 2.7.3. Basic treatment and rescue medication

All participants in the four groups will receive basic treatments under GOLD 2022, which are identical to the conventional treatment received by patients in the basic treatment group. It includes respiratory support (oxygen therapy or high-flow nasal therapy), and standard medications, such as bronchodilators (inhaled beta_2_-agonists or anticholinergics), corticosteroids (prednisone or prednisolone or budesonide or budesonide), antibiotics (based on the bacterial resistance pattern) and methylxanthines (theophylline or aminophylline). The treatment is individualized according to the actual situation of patients, and the standard medications can be used alone or in combination. In case of severe symptoms, such as sudden worsening of resting dyspnea, high respiratory rate, decreased oxygen saturation, even acute respiratory failure, or other emergency conditions, urgent or emergency care facilities will be prepared, including supplemental oxygen therapy, noninvasive mechanical ventilatory support, increasing doses and/or frequency of bronchodilators, corticosteroids and antibiotics, etc. The treatment utilized throughout the study will be recorded in detail in the case report form (CRF).

### 2.8. Outcome measurements

#### 2.8.1. Primary outcome

The primary outcome used in this study will be the differences between the Breathlessness, Cough, and Sputum Scale (BCSS) (37) before and after treatment. It is a kind of self-reported symptom severity scale with a total score of 12, which includes 3 symptoms (breathlessness, cough, and sputum). Every symptom generates a score ranging from 0 to 4, in which 0 means no symptoms, 1 means mild, 2 means moderate, 3 means obvious, and 4 means severe. The BCSS scores will be evaluated before randomization, after treatment, 5 weeks and 9 weeks.

#### 2.8.2. Secondary outcomes

##### 2.8.2.1. The COPD Assessment Test

The COPD Assessment Test (CAT) (38) is used for comprehensive assessment of disease-specific health status and quality of life, which is commonly used in routine practice. The CAT is an 8-item measurement tool, with a score ranges from 0 to 40, and the more severe the symptoms, the higher the score. It will be evaluated before randomization, after treatment, 5 weeks and 9 weeks.

##### 2.8.2.2. Pulmonary function test

Pulmonary function test (PFT) will be performed according to American Thoracic Society (ATS) (39, 40) guidelines with a Spirometry measurement maneuver. It will be conducted three times and then pick the maximum value when there is good consistency (the variation rate was less than 10%). Forced expiratory volume in 1 s (FEV_1_), FEV_1_pred (FEV_1_%), forced vital capacity (FVC) and the FEV_1_/FVC ratio are commonly used indicators in COPD (1). They can help to classify the severity of breathlessness, exercise limitation and health status impairment. Besides, FEV_1_ and FEV_1_% can determine the prognosis of COPD, and predict the risk of exacerbations, hospitalization, and death. The FEV_1_/FVC ratio less than 70% after using a bronchodilator confirms the presence of persistent airflow limitation, which is the “gold standard” for the diagnosis of COPD. Thus, FEV_1_, FEV_1_%, FVC, and FEV_1_/FVC will be evaluated before and after treatment.

##### 2.8.2.3. Arterial blood gas

Arterial blood gas (ABG) is an important indicator to evaluate the severity of exacerbation. Indicators including power of hydrogen (pH), partial pressure of oxygen (PaO_2_), and partial pressure of carbon dioxide (PaCO_2_) are recorded. The ABG will be collected before and after treatment by the clinical physicians in these tertiary hospitals. Once the blood is collected, it will be sent to Laboratory Medicine immediately.

##### 2.8.2.4. The 6-min walking test

The 6-min walking test (6MWT) will be conducted according to ATS guidelines (41). And the 6-Minute Walking Distance (6MWD) is monitored to assess exercise tolerance. It will be performed at a long, flat, and straight corridor, and subjects will be instructed to walk and cover as much distance as possible within 6 min. The 6MWT will be performed before and after completion of 7 days’ treatment.

##### 2.8.2.5. Hospital length of stay

Duration of hospitalization is calculated as the cumulative number of days from admission to discharge. It is helpful to judge the patient’s condition and the effectiveness of treatment.

##### 2.8.2.6. The number of exacerbations

Counting the number of exacerbations is beneficial to evaluate the efficacy of initial treatment, predict the prognosis, and then provide a guide for subsequent treatments (42). Thus, the number of exacerbations after discharge within 6 months due to AECOPD will be recorded at 25 weeks.

### 2.9. Safety and adverse events

The acupoints we select in this study are relatively safe. If an adverse effect occurs, the information of these adverse effects, such as the type, onset and end time, intensity and outcomes will be recorded in detail in CRF. Participants who suffer from adverse events will receive corresponding interventions. Besides, we will report severe adverse events immediately to the primary researchers and the ethics committees, and the affected participants will stop the clinical study.

### 2.10. Sample size

According to the previous literature (43), for the primary outcome BCSS, an improvement of 2.5 in basic treatment group is reported. Mean changes of the total score more than one represent a significant improvement (37). Based on expert opinions and our pilot study, we anticipated an improvement of 3.7 in the Eight Confluence points group, 3.5 in Xi-cleft and He-sea group and 2.7 in sham acupuncture group. One hundred and eleven patients per group would be needed as calculated by PASS 15.0 at a two-sided significance level of 5% and power of 80% in a 1:1:1:1 ratio. Estimating a 20% drop out rate, a total of 556 patients (139 patients per group) will need to be enrolled in this study.

### 2.11. Data management

All data will be recorded by outcome assessors at each site who have accepted specialized training before the study to ensure the consistency of manipulation in different centers. The training includes how to fill in the CRF, how to input data into Electronic Data Capture System (EDC), which is the electronic CRF established by Chinese Evidence-based Medicine Center, West China Hospital, Sichuan University. A quality control board will be organized to confirm the accuracy and consistency of the data. The data of each participant will be stored in the EDC system, and only data monitors can check the data. Researchers cannot modify or access the data until the data collection is completed.

### 2.12. Statistical analysis

The Chinese Evidence-based Medicine Center, West China Hospital, Sichuan University will complete the statistical analysis. The statistical analysis personnel will be blinded to the grouping. The data will be analyzed through SPSS 26.0 statistical software (IBM SPSS Statistics, IBM, Somers, NY, United States). The full analysis set (FAS) will be used to assess the validity of the study as a whole, and the per protocol set (PPS) will be used for sensitivity analysis. To compare the numerical variables among the four groups, the analysis of variance (ANOVA) test will be used when the data are normally distributed, and the Kruskal-Wallis test will be used for the analysis of skew distribution data. Categorical variables will utilize chi-square analysis. Mean ± standard deviation (SD) and 95% confidence intervals (CI) will be used to describe continuous data, while a frequency table and 95% CI will be used to describe categorical variables. Missing values will be addressed using the method of last observation carried forward (LOCF) and multiple-imputation.

### 2.13. Quality control

The study protocol has been reviewed and revised by experts in the fields of acupuncture, respiratory, and statistics. And all relevant members involved will be uniformly trained about the study protocol and standardized operation procedure before the study. Acupuncture treatment will be performed by acupuncturists who are licensed and have at least 3 years of acupuncture experience in clinical practice. Acupuncturists will receive standardized training on the location of acupoints and non-acupoints, as well as standardized procedures for acupuncture and sham acupuncture, to ensure consistency across different centers. And all original data obtained during this study will be checked repeatedly by outcome assessors. A quality control board will be organized to check the CRFs and operation procedure of acupuncture every 2 months, and then they will produce a report on the quality of the entire study process. The principal investigators will meet regularly to discuss and solve problems discovered during the observation period.

### 2.14. Ethics and dissemination

This study will be conducted under the standards of the International Coordinating Committee for Good Clinical Practice and the Declaration of Helsinki (44), and has been registered on the Chinese Clinical Trial Registry (CHICTR) platform. The protocol (completed on July 2, 2022, version 1) was approved by the Sichuan Regional Ethics Review of Committee on Traditional Chinese Medicine on 4 August, 2022 (Approval ID: 2022KL-068). Any important protocol modifications and other changes after the publication of this paper will be updated at the trial registration platform. Informed consent will be obtained from all participants before the start of the study. The privacy of participants will be protected and all original medical records and study data will be kept strictly confidential. The outcomes of the trial will be disseminated in an international peer-reviewed journal. Results of this trial will help evaluate the efficacy and safety of acupuncture with different acupoint combinations in the treatment of AECOPD.

## 3. Discussion

With the increasing prevalence of aging populations, the prevalence of COPD and COPD-related mortality are expected to rise in the coming decades, which leads to more serious economic, social, and medical burdens, and poses a major challenge to the global public health system (1, 8). Thus, it is time to make COPD care a public health priority in the twenty-first century (45). AECOPD can negatively affect health status, rates of hospitalization and readmission, and disease progression, making it an important event in COPD management (1). Therefore, how to effectively reduce acute exacerbations is particularly important for older adults. Studies have suggested that acupuncture may have a positive impact on AECOPD (17–19), however, the reliability of these findings is questionable due to methodological limitations such as small sample sizes and less rigorous randomization and blinding procedures. Currently, there is a dearth of larger studies that have assessed the efficacy and safety of acupuncture as a treatment modality for AECOPD during hospitalization. This study will provide solid evidence of acupuncture as an adjunctive treatment for patients with AECOPD. In addition, this is the first study to investigate the differences of different acupoint combinations in the treatment of AECOPD.

### 3.1. Grouping and acupoint selection

According to TCM theory, acupoints have local therapeutic effects, that is, each acupoint locates on a specific location is able to treat disorders in that area and nearby tissues and organs. Furthermore, the acupoints on the meridians can treat the diseases in the areas through which they pass. The Conception Vessel runs through the chest and can be used to treat lung diseases. Thus, we select Tiantu (RN22), Zhongwan (RN12), and Danzhong (RN17), which are located on or near the chest and belong to the Conception Vessel, as basic acupoints to ensure the effectiveness of acupuncture treatment. Acupoint combination, a vital element of the fundamental theory of acupuncture, has the potential to produce cooperative and synergistic effects, thereby enhancing the relevance of acupoints (46, 47). It is considered a key factor in improving clinical efficacy. Research indicates that the efficacy of acupoint combinations may vary (47). However, no studies have compared the efficacy of different acupoint combinations in treating AECOPD. Therefore, this study aims to investigate whether there are differences in the efficacy of two classical acupoint combinations in the treatment of AECOPD. AECOPD commonly affects the Lung Meridian, and acupoints located on this meridian are essential parts of acupoint prescription for acupuncture treatment of pulmonary symptoms, such as cough, dyspnea. Thus, except for the basic acupoints mentioned above, we will select Kongzui (LU6) and Chize (LU5), which are the specific acupoints of the Lung Meridian (the affected meridian), to enhance the therapeutic effect. Kongzui (LU6) and Chize (LU5) are the Xi-cleft acupoint and the He-sea acupoint of the Lung Meridian respectively, and the combination of the two acupoints may improve efficacy. In the Eight Confluence points group, we select Neiguan (PC6) and Gongsun (SP4) as additional acupoints, both of which are the Eight-confluent points. Neiguan (PC6) is the specific acupoints of the Pericardium Meridian of Hand-Jueyin, while Gongsun (SP4) is the specific acupoints of the Spleen Meridian of Foot-Taiyin, both acupoints are not on the lung meridian, but they are connected with the chest through the Yinwei Meridian and the Vital Meridian, respectively, so as to treat chest disorders. Based on TCM theory, the two acupoint combinations are considered classical modes for treating lung diseases. However, while one group of acupoints is located on the lung meridian, the other group is not. It is therefore necessary to investigate whether there are any discernible differences in therapeutic efficacy between the two groups. Such clarification would offer valuable insights for the study of acupoint combinations. The function of non-acupoints remains a topic of controversy, with certain studies indicating no discernible difference between acupoints and non-acupoints (48, 49). To more accurately assess the therapeutic efficacy of acupuncture and establish the specificity of acupoints, a sham acupuncture group was established as a reference to mitigate the potential placebo effect of acupuncture. The selection of the four non-acupoints utilized in this study was informed by expert consultation and prior research. Besides, we set up a basic treatment group (treat with routine western medicine only) as a reference to the acupuncture group and sham acupuncture group.

### 3.2. The multidimensional evaluation of acupuncture for treating AECOPD

This study aims to present compelling evidence that acupuncture is a safe and effective method for ameliorating symptoms, enhancing quality of life, improving exercise performance and pulmonary function, reducing hospitalization duration, and preventing re-acute exacerbations. Acute exacerbation of breathlessness, cough, or sputum is a defining feature of AECOPD (1). Consequently, we have chosen the BCSS scale as the primary outcome measure to evaluate the severity of symptoms. And there are only three closely related questions about breathlessness, cough or sputum in the scale, so it is easy to obtain daily changes in the total BCSS score throughout the entire treatment process and follow up directly. Additionally, we emphasize acupuncture’s therapeutic effects on other aspects of AECOPD, CAT is used to assess symptoms and quality of life comprehensively. Besides, objective outcomes are also selected to verify the efficacy of acupuncture and avoid subjective effects. For example, ABG is used to evaluate the blood gas conditions, PFT is used to evaluate lung functions, and 6MWT will be used to evaluate the exercise capacity. And the number of re-acute exacerbations will be utilized to determine the prognosis of AECOPD after receiving acupuncture. In addition, adverse reactions will be recorded in detail to demonstrate the safety of acupuncture.

### 3.3. The quality control criteria

To enhance the research’s quality, this trial employs central randomization, utilizing dynamic block randomization with a computer system’s central control to mitigate the impact of artificial or unknown factors. This method is a stringent randomization approach that guarantees appropriate allocation and concealment, thereby substantially enhancing research quality and efficiency. Additionally, the study protocol was meticulously designed by experts in acupuncture and respiratory fields and has been duly registered. Besides, we will restrict the number of acupoints and standardize the manipulation procedure, and a quality control board will be established to ensure the quality of the study. In a word, this is a multicenter trial with large sample size and strict design, which is expected to offer more insight into the clinical efficacy and safety of acupuncture on AECOPD. And the therapeutic effect of different acupoint combinations (Xi-cleft and He-sea point combination and Eight Confluence point combination) will be demonstrated, and then, explore and confirm the advantages and characteristics of acupoints combination.

In summary, the objective of this study is to ascertain the efficacy and safety of acupuncture as an adjunctive treatment for AECOPD patients. We hope this study will provide clinical guidance for clinicians and facilitate the use of acupuncture during the acute exacerbations of COPD and provide reference for future research.

### 3.4. Limitations

This study is subject to certain limitations. Specifically, in accordance with GOLD guidelines, COPD exacerbation symptoms typically persist for a duration of 7 to 10 days. Consequently, we opted to administer acupuncture treatment for a period of 7 days to enable the inclusion of a greater number of subjects. This abbreviated treatment duration may, however, underestimate the effectiveness of acupuncture. Additionally, given the inherent nature of acupuncture, it is not feasible to blind either acupuncturists or patients in the basic treatment group. Nevertheless, outcome assessors and statisticians will be fully blinded throughout the process and will remain unaware of the grouping. Thirdly, in accordance with Traditional Chinese Medicine (TCM) theory, numerous acupoint combination modes exist. However, due to constraints in manpower and material resources, only two modes have been studied thus far, necessitating further exploration in the future.

## Ethics statement

The studies involving humans were approved by the Sichuan Regional Ethics Review of Committee on Traditional Chinese Medicine. The studies were conducted in accordance with the local legislation and institutional requirements. The participants provided their written informed consent to participate in this study.

## Author contributions

FL, FZ, YS, MS, and CY performed the evaluation, participated in the conception and design of the trial, and drafted the manuscript. MC, XY, QL (Qian Li), JT, YLL, ZZ, FH, YYL, and DH participated in patient recruitment and data collection. LH, HT, CY, GX, and QL (Qin Luo) performed the acupuncture practice. SY and MS monitored the trial and case report forms. All authors have read and approved the final manuscript.

## Funding

This study was supported by the Innovation Team and Talents Cultivation Program of the National Administration of Traditional Chinese Medicine (No. ZYYCXTD-D-202003).

## Conflict of interest

The authors declare that the research was conducted in the absence of any commercial or financial relationships that could be construed as a potential conflict of interest.

## Publisher’s note

All claims expressed in this article are solely those of the authors and do not necessarily represent those of their affiliated organizations, or those of the publisher, the editors and the reviewers. Any product that may be evaluated in this article, or claim that may be made by its manufacturer, is not guaranteed or endorsed by the publisher.
