# Supplementary material for: Acupuncture as adjunctive therapy for patients with AECOPD: study protocol for a multicenter, randomized controlled trial
Source: Front Public Health. 2023 Oct 2;11:1235672. doi: 10.3389/fpubh.2023.1235672 (PMC10578458; doi:10.3389/fpubh.2023.1235672)
Supplement: Supplementary file 1 [file Data_Sheet_1.pdf]

Supplementary Material

Acupuncture as adjunctive therapy for patients with AECOPD: study protocol for a multicenter, randomized controlled trial

Chunyan Yang[1†], Mingsheng Sun[1†], Guixing Xu[1], Qin Luo[1], Liuyang Huang[1], Hao Tian[1], Siyao Gong[1], Qian Li[1], Xin Yu[1], Ming Chen[1], Dan Huang[1], Yilin Liu[1], Zhuo Zhou[1], Fengyuan Huang[1], Yunyu Liu[1], Juan Tang[1], Sha Yang[1*], Fang Zeng[1*], Fanrong Liang[1*]

*** Correspondence:**
Fanrong Liang: acuresearch@126.com; Fang Zeng: telepathy-zf@126.com; Sha Yang: yangsha@cdutcm.edu.cn.

1 Supplementary Table 1

SPIRIT 2013 Checklist: Recommended items to address in a clinical trial protocol and related documents*

Section/item	Item No	Description	
**Administrative information**			
Title	1	Descriptive title identifying the study design, population, interventions, and, if applicable, trial acronym	Page1
Trial registration	2a	Trial identifier and registry name. If not yet registered, name of intended registry	Page2
	2b	All items from the World Health Organization Trial Registration Data Set	NA
Protocol version	3	Date and version identifier	Page10
Funding	4	Sources and types of financial, material, and other support	Page13
	5a	Names, affiliations, and roles of protocol contributors	Page13

Roles and responsibilities	5b	Name and contact information for the trial sponsor	Page13
	5c	Role of study sponsor and funders, if any, in study design; collection, management, analysis, and interpretation of data; writing of the report; and the decision to submit the report for publication, including whether they will have ultimate authority over any of these activities	Page13
	5d	Composition, roles, and responsibilities of the coordinating centre, steering committee, endpoint adjudication committee, data management team, and other individuals or groups overseeing the trial, if applicable (see Item 21a for data monitoring committee)	Page13
Introduction			
Background and rationale	6a	Description of research question and justification for undertaking the trial, including summary of relevant studies (published and unpublished) examining benefits and harms for each intervention	Page2
	6b	Explanation for choice of comparators	Page3
Objectives	7	Specific objectives or hypotheses	Page3
Trial design	8	Description of trial design including type of trial (eg, parallel group, crossover, factorial, single group), allocation ratio, and framework (eg, superiority, equivalence, noninferiority, exploratory)	Page3
Methods: Participants, interventions, and outcomes			
Study setting	9	Description of study settings (eg, community clinic, academic hospital) and list of countries where data will be collected. Reference to where list of study sites can be obtained	Page3

Eligibility criteria	10	Inclusion and exclusion criteria for participants. If applicable, eligibility criteria for study centres and individuals who will perform the interventions (eg, surgeons, psychotherapists)	Page4
Interventions	11a	Interventions for each group with sufficient detail to allow replication, including how and when they will be administered	Page5
	11b	Criteria for discontinuing or modifying allocated interventions for a given trial participant (eg, drug dose change in response to harms, participant request, or improving/worsening disease)	Page8
	11c	Strategies to improve adherence to intervention protocols, and any procedures for monitoring adherence (eg, drug tablet return, laboratory tests)	Page4
	11d	Relevant concomitant care and interventions that are permitted or prohibited during the trial	Page8
Outcomes	12	Primary, secondary, and other outcomes, including the specific measurement variable (eg, systolic blood pressure), analysis metric (eg, change from baseline, final value, time to event), method of aggregation (eg, median, proportion), and time point for each outcome. Explanation of the clinical relevance of chosen efficacy and harm outcomes is strongly recommended	Pag8
Participant timeline	13	Time schedule of enrolment, interventions (including any run-ins and washouts), assessments, and visits for participants. A schematic diagram is highly recommended (see Figure)	Page3
Sample size	14	Estimated number of participants needed to achieve study objectives and how it was determined, including clinical and statistical assumptions supporting any sample size calculations	Page9
Recruitment	15	Strategies for achieving adequate participant enrolment to reach target sample size	Page4

Methods: Assignment of interventions (for controlled trials)

Allocation:

Sequence generation	16a	Method of generating the allocation sequence (eg, computer-generated random numbers), and list of any factors for stratification. To reduce predictability of a random sequence, details of any planned restriction (eg, blocking) should be provided in a separate document that is unavailable to those who enrol participants or assign interventions	Page5
Allocation concealment mechanism	16b	Mechanism of implementing the allocation sequence (eg, central telephone; sequentially numbered, opaque, sealed envelopes), describing any steps to conceal the sequence until interventions are assigned	Page5
Implementation	16c	Who will generate the allocation sequence, who will enrol participants, and who will assign participants to interventions	Page5
Blinding (masking)	17a	Who will be blinded after assignment to interventions (eg, trial participants, care providers, outcome assessors, data analysts), and how	Page5
	17b	If blinded, circumstances under which unblinding is permissible, and procedure for revealing a participant’s allocated intervention during the trial	Page5

Methods: Data collection, management, and analysis

Data collection methods	18a	Plans for assessment and collection of outcome, baseline, and other trial data, including any related processes to promote data quality (eg, duplicate measurements, training of assessors) and a description of study instruments (eg, questionnaires, laboratory tests) along with their reliability and validity, if known. Reference to where data collection forms can be found, if not in the protocol	Page9
	18b	Plans to promote participant retention and complete follow-up, including list of any outcome data to be collected for participants who discontinue or deviate from intervention protocols	Page4

Data management	19	Plans for data entry, coding, security, and storage, including any related processes to promote data quality (eg, double data entry; range checks for data values). Reference to where details of data management procedures can be found, if not in the protocol	Page9
Statistical methods	20a	Statistical methods for analysing primary and secondary outcomes. Reference to where other details of the statistical analysis plan can be found, if not in the protocol	Page10
	20b	Methods for any additional analyses (eg, subgroup and adjusted analyses)	NA
	20c	Definition of analysis population relating to protocol non-adherence (eg, as randomised analysis), and any statistical methods to handle missing data (eg, multiple imputation)	Page10
**Methods: Monitoring**			
Data monitoring	21a	Composition of data monitoring committee (DMC); summary of its role and reporting structure; statement of whether it is independent from the sponsor and competing interests; and reference to where further details about its charter can be found, if not in the protocol. Alternatively, an explanation of why a DMC is not needed	Page10
	21b	Description of any interim analyses and stopping guidelines, including who will have access to these interim results and make the final decision to terminate the trial	NA
Harms	22	Plans for collecting, assessing, reporting, and managing solicited and spontaneously reported adverse events and other unintended effects of trial interventions or trial conduct	Page9
Auditing	23	Frequency and procedures for auditing trial conduct, if any, and whether the process will be independent from investigators and the sponsor	Page10
Ethics and dissemination			

Research ethics approval	24	Plans for seeking research ethics committee/institutional review board (REC/IRB) approval	Page13
Protocol amendments	25	Plans for communicating important protocol modifications (eg, changes to eligibility criteria, outcomes, analyses) to relevant parties (eg, investigators, REC/IRBs, trial participants, trial registries, journals, regulators)	NA
Consent or assent	26a	Who will obtain informed consent or assent from potential trial participants or authorised surrogates, and how (see Item 32)	Page5
	26b	Additional consent provisions for collection and use of participant data and biological specimens in ancillary studies, if applicable	NA
Confidentiality	27	How personal information about potential and enrolled participants will be collected, shared, and maintained in order to protect confidentiality before, during, and after the trial	Page10
Declaration of interests	28	Financial and other competing interests for principal investigators for the overall trial and each study site	Page13
Access to data	29	Statement of who will have access to the final trial dataset, and disclosure of contractual agreements that limit such access for investigators	Page13
Ancillary and post-trial care	30	Provisions, if any, for ancillary and post-trial care, and for compensation to those who suffer harm from trial participation	NA
Dissemination policy	31a	Plans for investigators and sponsor to communicate trial results to participants, healthcare professionals, the public, and other relevant groups (eg, via publication, reporting in results databases, or other data sharing arrangements), including any publication restrictions	Page10
	31b	Authorship eligibility guidelines and any intended use of professional writers	NA

	31c	Plans, if any, for granting public access to the full protocol, participant-level dataset, and statistical code	NA
Appendices			
Informed consent materials	32	Model consent form and other related documentation given to participants and authorised surrogates	Page10
Biological specimens	33	Plans for collection, laboratory evaluation, and storage of biological specimens for genetic or molecular analysis in the current trial and for future use in ancillary studies, if applicable	NA

*It is strongly recommended that this checklist be read in conjunction with the SPIRIT 2013 Explanation & Elaboration for important clarification on the items. Amendments to the protocol should be tracked and dated. The SPIRIT checklist is copyrighted by the SPIRIT Group under the Creative Commons "Attribution-NonCommercial-NoDerivs 3.0 Unported" license.

2 Supplementary Table 2

Acupuncture treatment details based on the STRICTA checklist

Item	Detail
1. Acupuncture rationale	1a) Style of acupuncture
	- acupuncture
	1b) Reasoning for treatment provided, based on historical context, literature sources, and/or consensus methods, with references where appropriate
	- acupuncture treatment based on the theory of TCM, and clinical experience of experts in acupuncture and respiratory.
	1c) Extent to which treatment was varied
	- The treatment was not varied.
2. Details of needling	2a) Number of needle insertions per subject per session (mean and range where relevant)
	- 7.
	2b) Names (or location if no standard name) of points used (uni/bilateral)
	- Eleven points used: RN22 (unilateral); RN17 (unilateral); RN12 (unilateral); LU5 (bilateral); LU6 (bilateral); PC6 (bilateral); SP4 (bilateral).
	2c) Depth of insertion, based on a specified unit of measurement, or a particular tissue level
	- RN22: 0.3-0.5 cun, RN17: 0.3-0.5 cun, RN12: 0.3-0.5 cun, LU5: 0.5-1 cun, LU6: 0.5-1 cun, PC6: 0.5-1 cun, and SP4: 0.5-1 cun.
	2d) Response sought (e.g., de qi or muscle twitch response)

	- Needles will be manipulated with lifting, thrusting, twirling and rotating after inserting to achieve Deqi (a sensation including soreness, numbness, distention and heaviness).<br><br>2e) Needle stimulation (e.g., manual, electrical)<br><br>- manual<br><br>2f) Needle retention time<br><br>- Thirty minutes<br><br>2g) Needle type (diameter, length, and manufacturer or material)<br><br>- A disposable stainless steel acupuncture needle, 0.25mm × 13 mm and 0.25mm × 25 mm (Huatuo, Suzhou, Jiangsu, China).
3. Treatment regimen	3a) Number of treatment sessions<br><br>- Seven treatment sessions in acupuncture groups.<br><br>3b) Frequency and duration of treatment sessions<br><br>- Once a day, 30 minutes for each session.
4. Other components of treatment	4a) Details of other interventions administered to the acupuncture group (e.g., moxibustion, cupping, herbs, exercises, lifestyle advice)<br><br>- The standard treatment of AECOPD is based on Global Strategy for the Diagnosis, Management, and Prevention of Chronic Obstructive Lung Disease: the GOLD science committee report 2022.<br><br>4b) Setting and context of treatment, including instructions to practitioners and information and explanations to patients.<br><br>- Large tertiary class hospitals.

	- Participants will be informed about acupuncture treatment in the study as follows: "In this study, acupuncture for AECOPD will be used based on conventional pharmaceutical therapies."
5. Practitioner background	5) Description of participating acupuncturists (qualification or professional affiliation, years in acupuncture practice, other relevant experience)<br><br>- All acupuncturists participating in the study are qualified TCM physicians with a license in acupuncture and moxibustion, with at least 3 years of experience and were trained in the standard operating procedures of acupuncture for AECOPD. Thus, they can provide the same acupuncture treatment according to a predetermined protocol.
6. Control or comparator interventions	6a) Rationale for the control or comparator in the context of the research question, with sources that justify this choice<br><br>- We will set up two control groups, one of which will receive acupuncture on non-acupoints plus conventional treatment, and the other will receive only conventional treatment.<br><br>6b) Precise description of the control or comparator. If sham acupuncture or any other type of acupuncture-like control is used, provide details on items 1 to 3 above.<br><br>- The control group receiving sham acupuncture treatment, all patients will receive acupuncture on non-acupoints.<br><br>- Style of acupuncture: acupuncture<br><br>- Reasoning for treatment provided, based on historical context, literature sources, and/or consensus methods, with references where appropriate: acupuncture treatment based on literature research and clinical experience of experts in acupuncture and respiratory.<br><br>- Extent to which treatment was varied: the treatment was not varied.<br><br>- Number of needle insertions per subject per session (mean and range where relevant): 8.

	Names (or location if no standard name) of points used (uni/bilateral): 4 points used: NP-1 (bilateral), NP-2 (bilateral), NP-3 (bilateral), NP-4 (bilateral).<br><br>- Depth of insertion, based on a specified unit of measurement, or a particular tissue level: NP-1: 0.1-0.3 cun, NP-2: 0.1-0.3 cun, NP-3: 0.1-0.3 cun, and NP-4: 0.1-0.3 cun.<br><br>- Response sought (e.g., de qi or muscle twitch response): no.<br><br>- Needle stimulation (e.g., manual, electrical): manual.

Note: TCM: Traditional Chinese medicine; AECOPD: Acute exacerbation of chronic obstructive pulmonary disease; RN22: Tiantu; RN17: Danzhong; RN12: Zhongwan; LU5: Chize; LU6: Kongzui; PC6: Neiguan; SP4: Gongsun; GOLD: Global initiative for chronic obstructive lung disease; NP: Non-acupoint.
